# Anti-Aging Constituents from *Pinus morrisonicola* Leaves

**DOI:** 10.3390/molecules28135063

**Published:** 2023-06-28

**Authors:** Ta-Wei Liu, Sui-Wen Hsiao, Chi-Ting Lin, George Hsiao, Ching-Kuo Lee

**Affiliations:** 1 School of Pharmacy, Taipei Medical University, 250 Wu Xin Street, Taipei 11031, Taiwan; d301110010@tmu.edu.tw; 2 Ph.D. Program in Drug Discovery and Development Industry, College of Pharmacy, Taipei Medical University, 250 Wu Xin Street, Taipei 110301, Taiwan; d343106004@tmu.edu.tw; 3 Graduate Institute of Pharmacognosy, Taipei Medical University, 250 Wu Xin Street, Taipei 11031, Taiwan; m303105005@tmu.edu.tw; 4 Graduate Institute of Medical Sciences, College of Medicine, Taipei Medical University, Taipei 11031, Taiwan; geohsiao@tmu.edu.tw; 5 Department of Pharmacology, School of Medicine, Taipei Medical University, Taipei 11031, Taiwan; 6 Department of Chemistry, Chung Yuan Christian University, Zhongbei Road, Zhongli District, Taoyuan 32023, Taiwan

**Keywords:** *Pinus morrisonicola* Hayata, pinaceae, anti-aging, HT-1080 cells, cosmetics

## Abstract

*Pinus morrisonicola* Hayata is a unique plant species found in Taiwan. Previous studies have identified its anti-hypertensive, anti-oxidative, and anti-inflammatory effects. In this study, a bioactivity-guided approach was employed to extract 20 compounds from the ethyl acetate fraction of the ethanol extract of *Pinus morrisonicola* Hayata’s pine needles. The anti-aging effects of these compounds were investigated using HT-1080 cells. The structures of the purified compounds were confirmed through NMR and LC-MS analysis, revealing the presence of nine flavonoids, two lignans, one coumarin, one benzofuran, one phenylic acid, and six diterpenoids. Among them, PML18, PML19, and PML20 were identified as novel diterpene. Compounds **3**, **4**, and **5** exhibited remarkable inhibitory effects against MMP-2 and showed no significant cell toxicity at 25 μM. Although the purified compounds showed lower activity against Pro MMP-2 and Pro MMP-9 compared to the ethyl acetate fraction, we speculate that this is the result of synergistic effects.

## 1. Introduction

The skin is considered the largest organ in the human body. Skin aging is a complex process that can be caused by either intrinsic natural aging or external factors [1]. Intrinsic aging is associated with decreased mitotic activity, increased duration of the cell cycle and migration time, which can result in poor wound healing and metabolism. These effects can result in a reduction in dermis thickness, a decrease in the number of fibroblasts, and impaired functionality of sebaceous and sweat glands. Additionally, they lead to a reduction in the microvasculature in the skin, which lowers skin vascular reactivity, temperature regulation, and nutrient supply, resulting in pale or sallow skin [2]. The most important environmental factors contributing to skin aging are UV radiation and smoking, which increase the expression of matrix metalloproteinases (MMPs) in the skin [3,4]. MMPs, are responsible for degrading extracellular matrix proteins, such as collagen, fibronectin, elastin, and proteoglycans [5]. UV radiation-induced MMP expression plays a crucial role in the mechanism of photoaging through a series of signaling pathways [6]. MMPs are regulated by the transcription factor AP-1, which is significantly increased after UV exposure, resulting in increased mRNA and protein expression of MMPs. Increased level of MMPs leads to excessive degradation of the extracellular matrix, causing structural breakdown of the skin, wrinkles, and loss of elasticity, which in turn results in aging [6,7,8].

*Pinus morrisonicola* Hayata is a plant in the family of Pinaceae and the genus of Pinus. It is an endemic species in Taiwan and is mainly distributed in mountainous areas at altitudes of 300 to 2000 m, and is a tall evergreen tree with a trunk height of up to 30 m and a diameter of 1.2 m. The bark is dark gray and has a scale-like cracked appearance. The leaves are needle-shaped, with five needles in a bundle, and are 4 to 10 cm long [9]. The main components of *P. morrisonicola* are flavonoids (chrysin, apigenin) [10], stilbenes (pinosylvin and its derivatives) [11], terpenoids (pinene, terpinene) [12], and steroids (β-sitosterol) [13]. A previous study has demonstrated that *P. morrisonicola* extract exhibits good cell toxicity against GBM8901 glioblastoma cells [14], and the potential for anti-hypertensive, anti-oxidative, and anti-inflammatory effects [15,16,17]. Based on its extracts’ significant antioxidant and anti-inflammatory properties and the fact that flavonoids, the main component of *P. morrisonicola*, have shown anti-aging effects [18], it is expected to find potential anti-aging compounds from *P. morrisonicola*. Therefore, the purpose of this study is to isolate potential anti-aging compounds from *P. morrisonicola*.

## 2. Results

### 2.1. Bioasasay-Guided Compound Isolated from Pinus morrisonicola Hayata Leaves

**Figure 1:**
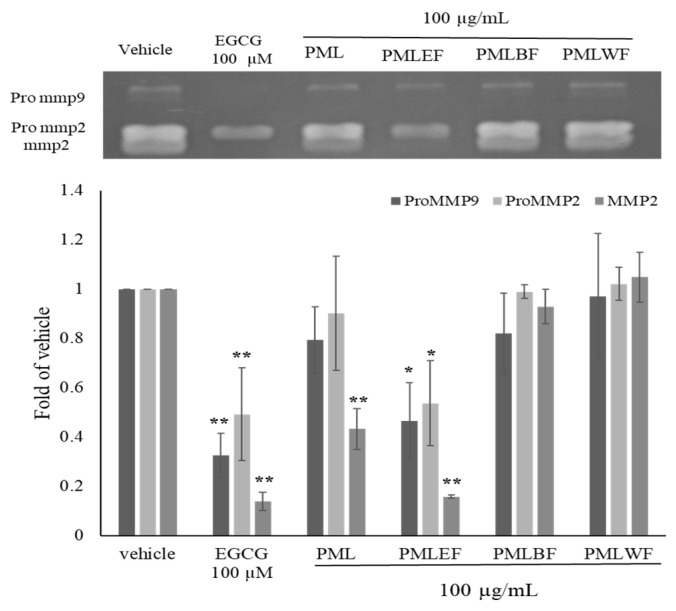
Activity effects of crude extract and three layers on MMP-2, Pro MMP-2 and Pro MMP-9 in HT-1080 human fibrosarcoma cell. Crude extraction (PML), Ethyl acetate layer (PMLEF), *n*-Butanol layer (PMLBF), and Water layer (PMLWF). *p*-values were derived from one-way ANOVA with Student-Newman-Keuls Tests. * *p*-value < 0.05, ** *p*-value < 0.01.

The leaves of *Pinus morrisonicola* Hayata were extracted with ethanol in a 10-times volume to obtain the crude extract (PML), which was then liquid-liquid partitioned to obtain the ethyl acetate layer (PMLEF), *n*-butanol layer (PMLBF), and water layer (PMLWF). The zymography method was then employed to assess the inhibitory activities of crude extract and three layers against Pro MMP-9, Pro MMP-2, and MMP-2. At 100 g/mL, PML showed significant inhibitory effects only on MMP-2 (0.43 ± 0.10), while PMLEF exhibited the best inhibitory activity against Pro MMP9 (0.47 ± 0.16), Pro MMP2 (0.54 ± 0.17), and MMP2 (0.16 ± 0.01) (Figure 1). Based on the significant cell activity of PMLEF, further isolation of its active components will be conducted.

### 2.2. Bioactive Compound Isolated from the Ethyl Acetate Layer (PMLEF) of Pinus morrisonicola Hayata Leaves

**Figure 2:**
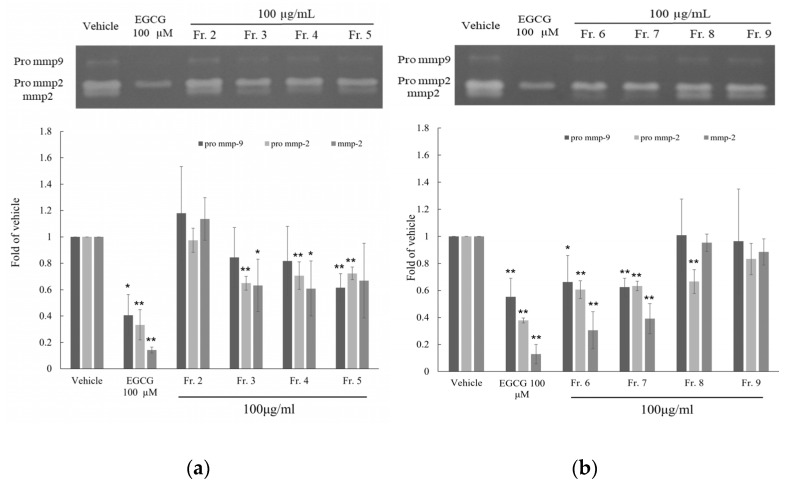
Activity effects of PMLEF fractions (Fr.2~Fr.9) on MMP-2, Pro MMP-2 and Pro MMP-9 in HT1080 human fibrosarcoma cell, and use EGCG as positive control. (**a**) PMLEF Fr.2~5 (**b**) PMLEF Fr.6~9 indicates significant differences from the vehicle. *p*-values were derived from one-way ANOVA with Student-Newman-Keuls Tests. * *p*-value < 0.05, ** *p*-value < 0.01.

Ten fractions were obtained through column chromatography from PMLEF, and these fractions were further screened for activity using HT-1080 cells. The results showed that Fr.5 (0.62 ± 0.11), Fr.6 (0.66 ± 0.20), and Fr.7 (0.62 ± 0.06) exhibited better inhibitory activity against Pro MMP-9, while Fr.6 and Fr.7 showed better inhibitory activity against Pro MMP-2 and MMP-2, with fold of vehicle values of 0.61 ± 0.07,0.31 ± 0.14 and 0.63 ± 0.04, 0.39 ± 0.11, respectively. (Figure 2) In addition, Fr.3 and Fr.4 also showed good inhibitory effects against Pro MMP-2 and MMP-2, especially with inhibition rates of 0.71 ± 0.10, 0.61 ± 0.21 and 0.72 ± 0.05, 0.67 ± 0.28. (Figure 2) Based on the activity-guided strategy, the subsequent purification prioritized Fr.3 to Fr.7 as the primary target for separation.

**Figure 3:**
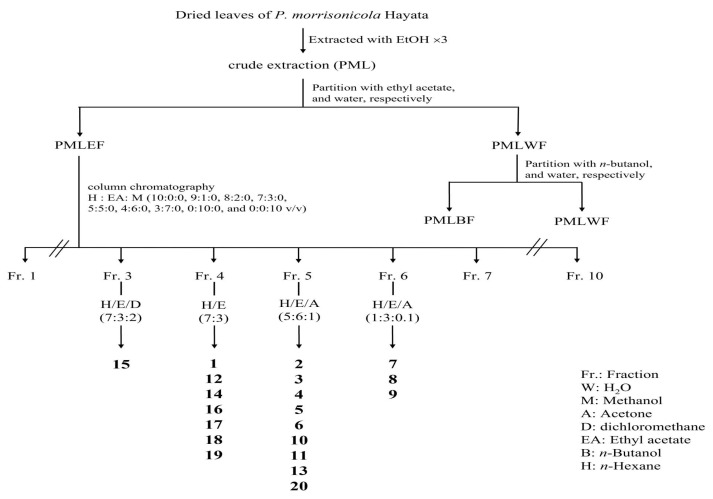
Flow chart of isolation procedure of subfractions of PMLEF.

**Figure 4:**
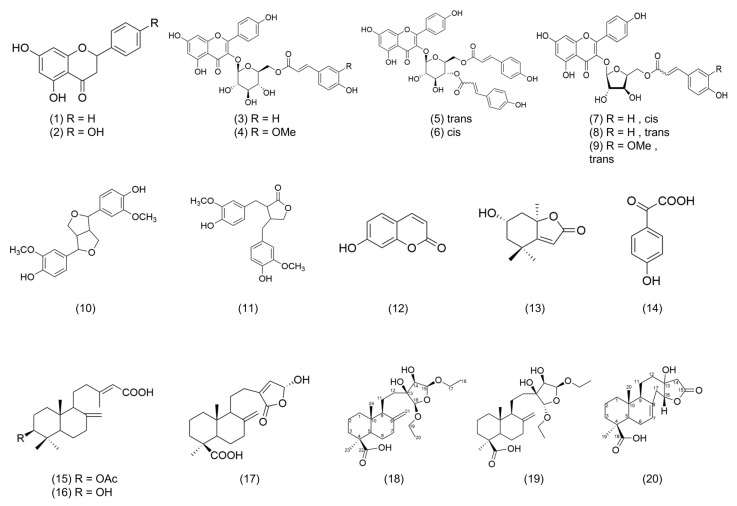
The structures of compounds **1**–**20** from the leaves of *Pinus morrisonicola* Hayata.

A total of 20 compounds were isolated from Fr.3~Fr.7 of PMLEF, and the structures were confirmed through NMR, MS, and literature comparison, as shown in Figure 3. Among them, PML4, PML18, PML19, and PML20 were determined as new compounds. The compounds are classified and named as follows (Figure 4): nine flavonoids, including chrysin (**1**) [19], apigenin (**2**) [20], Kaempferol 3-*O*-(6″-*O*-E-coumaroyl)-β-d-glucopyranoside (**3**) [21], Kaempferol 3-*O*-(6″-*O*-E-feruloyl)-β-d-glucopyranoside (**4**) [21], Kaempferol 3-*O*-(3″,6″-di-*O*-E-*p*-coumaroyl)-β-d-glucopyranoside (**5**) [22], Stenopalustrosides C (**6**) [22], Kaempferol 3-*O*-(5″-*O*-Z-*p*-coumaroyl)-α-l-arabinofuranoside (**7**) [23], Kaempferol 3-*O*-(5″-*O*-E-*p*-coumaroyl)-α-l-arabinofuranoside (**8**) [23], and Kaempferol 3-*O*-(5″-*O*-E-feruloyl)-α-l-arabinofuranoside (**9**) [23]. There are also two lignans, pinoresinol (**10**) [24] and matairesinol (**11**) [25], and one coumarin, 7-hydroxycoumarin (**12**) [26], as well as one benzofuran, loliolide (**13**) [27], and one phenylic acid, benzeneacetic acid (**14**) [28]. Additionally, there are six diterpenoids, including 3-acetoxylabda-8(20),13-diene-15-oic acid (**15**) [29], 3-hydroxylabda-8(20),13-diene-15-oic acid (**16**) [29], 13-labdadien-16, 15-olid-18-oic acid (**17**) [30], PML18, PML19, and PML20.

#### 2.2.1. Structure Analysis of PML18, PML19

**Figure 5:**
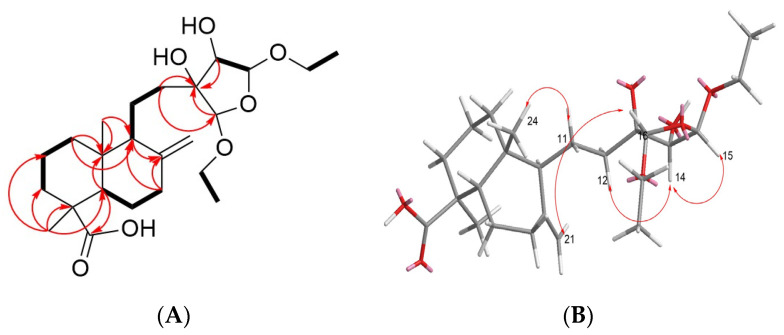

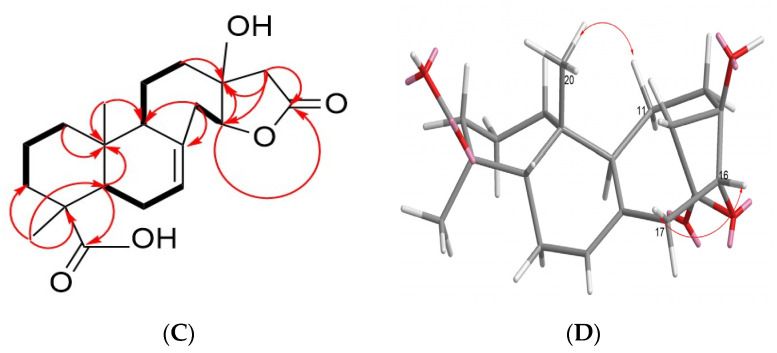
Selected 2D NMR (Chloroform-d) correlations for **18**, **19**, and **20**. (**A**) **18** COSY: ―; HMBC: (H→C). (**B**) **19** NOESY (**C**) **20** COSY: ―; HMBC: → (H→C). (**D**) **20** NOESY.

Compound **18** was obtained as a colorless oil from Fr. 4 after purification. Its molecular formula was deduced to be C_24_H_40_O_7_ based on the high-resolution electrospray ionization-tandem mass spectrometry (HRMS) [M-H]^-^ ion at m/z 439.2698 (cal. 439.2696) in negative mode, along with the ^13^C-NMR and DEPT spectra, which indicated a degree of unsaturation of 5 (Figures S2 and S16). The DEPT-NMR spectrum revealed the presence of four methyl groups, ten methylene groups, five methine groups, and five quaternary carbons (Figure S16). One quaternary carbon at δc 81.5 appeared downfield, suggesting its connection to a hydroxyl group. (Table 1) Two ethoxy groups were identified based on the ^1^H-^1^H COSY spectrum, where two proton signals at δ_H_ 1.18 were correlated with those at δ_H_ 3.51, 3.77 and δ_H_ 3.45, 3.74, respectively (Figure 5A and Figure S3). The proton at δ_H_ 4.82, 4.96, which linked the two ethoxy groups, also showed a downfield shift, likely due to the influence of the connecting oxygen (Figure 5A and Figure S3). The ^13^C-NMR spectrum showed a terminal double bond at δc 107.6, which was revealed by the ^1^H-^13^C HSQC spectrum to be correlated with the protons at δ_H_ 4.55, 4.80. (Figure S5, Table 1) A carboxylic acid carbon at δc 183.3 was shown on a ^13^C-NMR spectrum (Table 1). The degree of unsaturation of 5 suggested the presence of two six-membered rings and one five-membered ring after subtracting one carboxylic acid and one double bond, indicating PML **18** is a diterpene structure.

Based on the COSY experiment, the protons at δ_H_ 1.83, 1.96 (H-6) and at δ_H_ 1.28 (H-5), δ_H_ 1.84, 2.36 (H-7) were found to be correlated; the protons at δ_H_ 1.52 (H-2) were correlated with δ_H_ 1.07, 1.81 (H-1) and δ_H_ 1.01 (H-3) (Figure 5A and Figure S3); The protons at δ_H_ 1.51 (H-9) was correlation between δ_H_ 1.47, 1.83 (H-11); and the proton δ_H_ 4.96 (H-15) was correlated to δ_H_ 3.91 (H-14) (Figure 5A and Figure S3). In the HMBC experiment, the methyl group at δ_H_ 1.20 was found to be correlated with the carbons at δc 44.4 (C-4), 38.2 (C-3), and 56.6 (C-5) (Figure 5A and Figure S6); oxymethylene group δ_H_ 3.51, 3.77 (H-17) was found to be correlated with the carbons at δc 15.4, 109.2 (C-15), δ_H_ 3.45, 3.74 (H-19) was found to be correlated with the carbons at δc 15.4,107.3 (C-16); δ_H_ 3.91 (H-14) was found to be correlated with the carbons at δc 81.5 (C-13), 109.2 (C-15), 107.3 (C-16) (Figure 4A and Figure S6). According to the above NMR analysis, the planar structure of compound **18** is shown in Figure 4. The methyl group (H-24) on C10 of compound **18** is affected by the isotropic acid on C4 at high magnetic fields (δ_H_ 0.75) (Figure S1). The δ_H_ 0.57 determined from NOESY spectra correlates with δ_H_ 1.83 at C-11, indicating that -COOH and H-9 are in the β-orientation (Figure S4). The proton δ_H_ 3.91 of H-14 is correlated with δ_H_ 1.44, 1.75 of H-12 and δ_H_ 4.96 of H-15, indicating that the hydroxyl group of C-13, C-14 and the ethoxy group of C-15 are all in the β-direction (Figure S4). Therefore, the three-dimensional structure of compound **18** is shown in Figure 4.

Compounds **19** and **18** are stereoisomers, and their ^1^H NMR spectra are very similar. The only difference is that the H-12 (δ_H_ 1.31, 1.96) and double bonds (δ_H_ 4.64, 4.84) of compound **19** are slightly different from those of compound **18**. The NOESY experiment found that H-16 (δ_H_ 4.83) of compound **19** was related to the double bond but not **18** (Figure 5B, Figures S1, S4 and S7). Therefore, it can be determined that the ethoxy group at C-16 in PML**18** is a β-form, while ethoxy group at C-16 in PML19 (δ_H_ 4.83) is an α-form. Therefore, the structures of compounds **18** and **19** were determined as 15β,16β-diethoxy,13,14-dihydroxy-labd-8(21)-en-22-oic acid (**18**) and 15β,16α-diethoxy,13,14-dihydroxy-labd-8(21)-en-22-oic acid (**19**).

#### 2.2.2. Structure Analysis of PML20

PML **20** was purified from Fraction 5 and obtained as a colorless oil. HRMS in negative mode gave [M-H]^−^ m/z of 347.1867 (cal. 347.1859), indicating the molecular formula of C_20_H_28_O_5_ with a degree of unsaturation of 7 (Figure S18). NMR spectrum showed one carboxylic acid at δ_C_ 178.9 (C-18), one ester group at δ_C_ 175.7 (C-15), and one double bond signal at δ_H_ 5.68, suggesting the presence of four rings (Figures S10 and S11, Table 1). The ^1^H-NMR, ^13^C-NMR and DEPT spectra indicated that compound **20** has two methyls, eight methylene, four methine, and six quaternary carbons. ^1^H-^1^H COSY spectrum showed that δ_H_ 2.20 and 2.52 (H-6) were correlated to δ_H_ 1.39 (H-5) and 5.68 (H-7); δ_H_ 1.87 (H-9) was correlated to δ_H_ 1.45, 1.75 (H-11), and δ_H_ 1.87 (H-12), while δ_H_ 4.18 (H-16) was correlated with δ_H_ 2.30, 2.40 (H-17) (Figure 5C and Figure S12). HMBC experiment showed that δ_H_ 4.18 was correlated to ester group δ_C_ 175.7 (C-15) and quaternary carbon δ_C_ 79.2 (C-13), and C-13 was correlated to δ_H_ 4.18 (H-16), δ_H_ 2.35, 2.81 (H-14), δ_H_ 1.87, 2.07 (H-12), and δ_H_ 1.45, 1.75 (H-11). Lastly, the quaternary carbon of the double bond (C8, δ_C_ 134.6) was correlated with δ_H_ 2.30, 2.40 (H-17) and 1.45 (H-11) (Figure S15).

In terms of stereochemistry, the proton at δ_H_ 0.75 (C-20) was relatively upfield, suggesting its axial orientation was influenced by the carboxylic acid at C-18, as it did not show any correlation with δ_H_ 1.20, but with δ_H_ 1.45, 1.75 in the NOESY spectrum. (Figure 5D, Figure S10 and S13) Therefore, it was confirmed that H-20 and -COOH were in the axial direction (β-orientation), while H-9 was located in the equatorial direction. Furthermore, δ_H_ 4.18 was also observed to be in the β-orientation, as it showed correlation with δ_H_ 1.45 and δ_H_ 1.87 in the NOESY spectrum (Figure 5D and Figure S13). Thus, the structure of compound **20** was determined to be Morrisonicolene.

### 2.3. Anti-Aging Activity Test of Flavonoid Compounds

**Figure 6:**
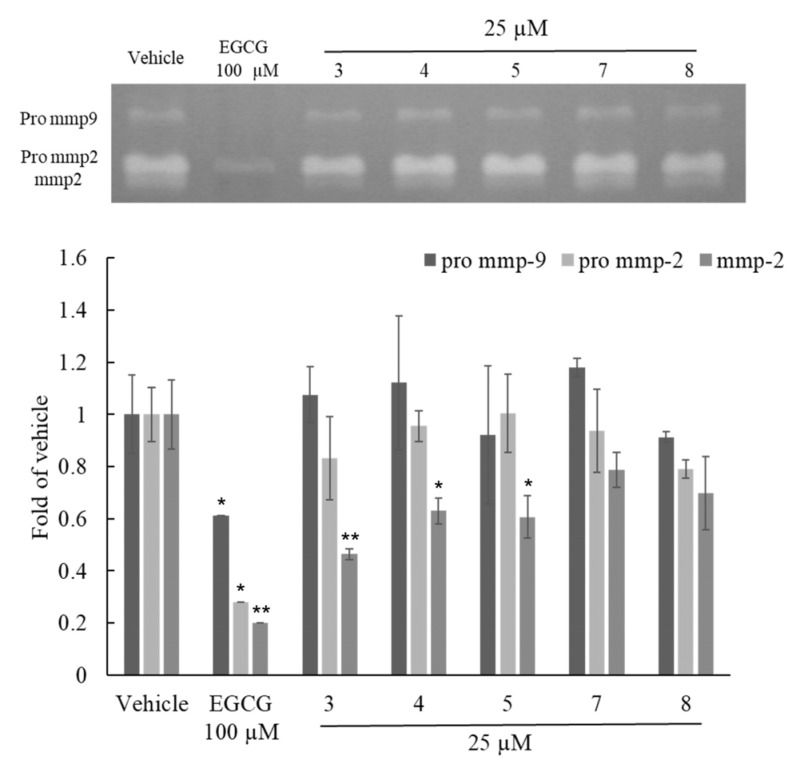
Activity effects of pure compounds from *P. morrisonicola* on MMP-2, Pro MMP-2 and Pro MMP-9 in HT1080 human fibrosarcoma cell. *p*-values were derived from one-way ANOVA with Student-Newman-Keuls Tests. * *p*-value < 0.05, ** *p*-value < 0.01.

Due to the potential anti-aging effects of flavonoids, our focus was on screening the flavonoid compounds in the active fraction for their anti-aging activity [18]. The cell viability of compounds **3**, **4**, **5**, **7**, and **8** was evaluated through MTT assay at a concentration of 25 μM for 24 h, showing no cytotoxicity. Subsequently, the effects of these flavonoid compounds **3**, **4**, **5**, **7** and **8** at a concentration of 25 μM on MMP-2, Pro MMP-2, and Pro MMP-9 were evaluated using zymography. As per the results, as shown in Figure 6, compounds **3**, **4**, and **5** exhibited significant inhibitory effects on MMP-2, with inhibition rates of 0.46 ± 0.05, 0.63 ± 0.08, and 0.60 ± 0.07, respectively. Among them, compound **3** demonstrated a particularly remarkable inhibitory effect. None of the five compounds exhibited significant inhibitory effects on Pro MMP-2 and Pro MMP-9. (Figure 6) The inhibitory activity on MMPs indicated the potential anti-aging effect of these compounds.

## 3. Discussion

In this study, a total of 20 compounds were isolated and purified from PMLEF by active fractionation method. Apart from compounds **1** and **2**, the remaining constituents were discovered for the first time in *Pinus morrisonicola* Hayata [10]. Compounds **8** and **9** have been identified in other *Pinus* species [23]. In our study, we revealed flavonoids with sugar moieties and coumaroyl group or feruloyl group, besides the commonly reported hydroxy group-containing flavonoids in *P. morrisonicola*. Additionally, our findings demonstrated the presence of diterpenes, which is consistent with our previous finding on *Pinus taiwanensis* Hayata [31], while previous studies predominantly reported monoterpenes in *Pinus* species.

Both PML and PMLEF exhibited significant inhibitory effects on MMP-2, Pro MMP-2, and Pro MMP-9 in the Zymographic assay. However, no compound displayed significant inhibition of Pro MMP-2 and Pro MMP-9, which contradicted the results obtained from fractions 3–7 of PMLEF. The purified compounds **3**, **4**, **5**, **7**, and **8** obtained from the activity-guided fractionation did not show comparable effects to PML or PMLEF. Hence, we speculate that PML possesses multiple compounds working synergistically to inhibit Pro MMP-2 and Pro MMP-9. In the case of MMP-2, compounds **3**, **4**, and **5** demonstrated significant effects (Figure 6); this suggests that glucopyranoside (compounds **3**, **4**, **5**) exhibits better activity than arabinoside (compounds **7**, **8**). The result in Figure 6 indicated that both compounds **7** and **8** mildly inhibited MMP-2 activity, without obvious differences between them. Considering the structural difference between compounds **7** and **8**, which lies in the cis or trans configuration of the double bond, it can be concluded that the orientation of the double bond does not affect their inhibitory activity against MMP-2. Furthermore, compound **3** showed stronger inhibition of MMP-2 compared to compound **4**, while the structural dissimilarity between compounds **3** and **4** lies only in the substitution of the coumaroyl group with the feruloyl group at the 3‴ position. Thus, the presence of a methoxy group at the 3‴ position is likely to decrease the inhibitory effect on MMP-2 (Figure 4 and Figure 6).

According to the MMPs assay, the EA layer of PML and its fractions showed remarkable activity on Pro MMP-9, Pro MMP-2 and MMP-2. However, compounds **3**, **4** and **5** only revealed an inhibitory effect on MMP-2. Therefore, we considered the EA layer of PML or its fraction to have more potential to serve as anti-aging cosmetics owing to the multi-component effect. In the previous study, chrysin could increase collagen I secretion and decrease the degradation of collagen I to repair oxidation damage. In addition, chrysin has been presented to inhibit melanin synthesis by reducing tyrosinase activity and suppressing the expression of melanogenic proteins [32] (Table 2). Choi et al. indicated that apigenin reduced the expression of collagenase [33], and Park et al. demonstrated that apigenin exhibited anti-aging and anti-inflammatory effects through the inhibition of nitric oxide (NO) production and cytokine expression in RAW264.7 cells and inhibited the expression of high-affinity IgE receptor and cytokines in RBL-2H3 cells [34] (Table 2). Loliolide reduces the activity of senescence-associated β-galactosidase (SA-β-gal) and decreases the levels of p21 protein, exerting an inhibitory effect in human dermal fibroblasts [35] (Table 2). Moreover, loliolide exhibits significant antioxidant and anti-inflammatory activities, as well as photoprotective effects, by improving collagen synthesis, reducing intracellular reactive oxygen species (ROS) levels, and inhibiting apoptosis in UVB-irradiated human keratinocytes and the expression of matrix metalloproteinases. It also reduces ROS, NO, lipid peroxidation, and cell death in UVB-irradiated zebrafish [36] (Table 2). Pinoresinol showed the antioxidant and anti-UV radiation through SPF value, UV absorption capacity, and the DPPH assay [37] (Table 2). At last, PML4, PML5 and PML11 all demonstrated antioxidants in DPPH radical-scavenging activity [38,39,40] (Table 2). Based on our research and previous study, we believe that PML and PMLEF might have the potential to be developed as versatile cosmetic ingredients.

## 4. Materials and Methods

### 4.1. Chemicals and Reagents

Methanol (ACS Grade), ethyl acetate (ACS Grade), dichloromethane (ACS Grade), and *n*-hexane (ACS Grade) were purchased from Mallinckrodt (St. Louis, MO, USA). *n*-Butanol (ACS Grade) was purchased from J. T. Baker. Methanol-d4 (CD_3_OD), acetone-d6 (CD_3_COCD_3_), Dimethyl sulfoxide-d6 ((CD_3_)_2_SO) and chloroform-d (CDCl_3_) were obtained from Merck (Darmstadt, Germany). Penicillin-streptomycin solution (PS), thiazolyl blue tetrazolium bromide (MTT), and Dulbecco’s Modified Eagle’s Medium-high glucose (DMEM) were purchased from Sigma-Aldrich (St. Louis, MO, USA). Fetal bovine serum (FBS) was obtained from SAFC Biosciences (Victoria, Australia).

### 4.2. Plant Material

*Pinus morrisonicola* Hayata leaves were collected from Forestry Research Institute in Taipei, Taiwan (coordinates 25°1′52″ N; 121°30′37″ E) and identified by Dr. Sheng-You Lu of Taiwan Forestry Research Institute.

### 4.3. Extraction and Isolation

#### 4.3.1. Extraction and Partition

A total of 9.8 kg of dried *Pinus morrisonicola* Hayata leaves were soaked in 10 times volume of ethanol and extracted three times. The resulting extract was concentrated under evaporator to obtain a crude extract (692 g). The crude extract was then partitioned with water, ethyl acetate, and *n*-butanol, and obtained an ethyl acetate layer (293 g), a *n*-butanol layer (297 g), and a water layer (102 g).

#### 4.3.2. Column Chromatography

A total of 220 g of the ethyl acetate layer were subjected to column chromatography using a silica gel column. The mobile phases used were *n*-hexane: ethyl acetate: methanol ratio from 10:0:0, 9:1:0, 8:2:0, 7:3:0, 5:5:0, 4:6:0, 3:7:0, 0:10:0, and 0:0:10 (*v*/*v*). The elution process yielded 10 fractions.

All compounds were obtained from Fr. 3–6 by normal phase, semi-preparative high-performance liquid chromatography (HPLC) (HITACHI L-7100, Hitachi, Tokyo, Japan) coupling with Bischoff Refractive Index (RI) detector for detection. Phenomenex Luna semi-preparative column (250 × 10 mm) (Phenomenex, Torrance, CA, USA) was performed, and the flow rate was 3 mL/min and eluted with *n*-hexane, dichloromethane, ethyl acetate and acetone (Figure 3).

#### 4.3.3. NMR and LC-MS Analysis

Methanol-d4, acetone-d6, dimethyl sulfoxide-d6 and chloroform-d were used as the deuterated solvent to obtain ^1^H, ^13^C, ^1^H–^1^H correlated spectroscopy (COSY), ^1^H–^1^H Nuclear Overhauser Effect Spectroscopy (NOESY), heteronuclear single quantum coherence spectroscopy (HSQC) and heteronuclear multiple-bond correlation spectroscopy (HMBC) NMR spectra on Bruker AV-300 MHz and AV-500 MHz spectrometers (Bruker, Rheinstetten, Germany). Orbitrap QE Plus (ESI-MS) (Thermo Fisher Scientific, Waltham, MA, USA) was used for purified compound molecular weight determination, and data were processed by Xcalibur (version 2.2).

### 4.4. Cell Line and Culture

HT-1080 cells (purchased from ATCC number: CCL-121) were maintained in RPMI-164 medium supplemented with 10% FBS, 1% PSQ (2 mM,100 U/mL Penicillin and 100 μg/mL Streptomycin, l-Glutamine) at 37 °C and 5% CO_2_ in an incubator. Cells were cultured in RPMI-1640 medium containing 0.5% FBS before use.

### 4.5. MTT Cell Viability Assay

HT-1080 cells were seeded by 5 × 10^5^ cells/mL per well in 24-well plates for 24 h, and treated with crude extract or pure compound for another 22 h. Then, cells were cultured with MTT solution for further 2 h. After that, supernatants were removed and 400 μL DMSO was added to the plate. Mixtures were transferred to 96-well plate and detected by ELISA reader (MRX microplate reader, Vodickova, Czech Republic) under the wavelength of 550 nm. Cell viability was calculated as follows:

(Treating absorbance value)/(Resting absorbance value) × 100%(1)

### 4.6. Zymography

HT-1080 cells were placed at a density of 5 × 10^5^ cells/mL in 24-well plate and incubated at 37 °C for 24 h to allow attachment. After treatment with samples, the cells were incubated for an additional 24 h at 37 °C. The reactions were then terminated and cell supernatant was mixed with sample loading dye in a 1:1 volume ratio and thoroughly mixed. The mixture was then subjected to electrophoresis on a 10% polyacrylamide gel (containing 1% gelatin) in running buffer at 130 V and 90 mA. The gel was then washed two times with 2.5% Triton X-100 at room temperature for 30 min each. The gel was then incubated in reaction buffer at 37 °C for 24 h, and further 30 min to immobilize the proteins on the gel by fixing solution. The gel was then stained uniformly using Brilliant Blue G-Colloidal Concentrate, and destained using destain solution to optimize the results. Finally, the gel was photographed using CCD in an imaging analysis system (Vilber Lourmat, France), and the image was analyzed using imaging analysis software (Bio-1D version 99). The brightness of the vehicle was used as the reference value of 1, and the brightness of the other bands was expressed in relative multiples.

### 4.7. Statistical Analyses

The data results of this experiment are expressed as Mean ± SD. Statistical analysis was performed using One Way ANOVA followed by the Student-Newman-Keuls Test. A *p* value less than 0.05 indicates a significant difference.

## 5. Conclusions

This study investigated the biological activities and chemical composition of *Pinus morrisonicola* Hayata pine needles. PMLEF exhibited significant inhibitory effects on MMP-2, Pro MMP-2, and Pro MMP-9. Therefore, we consider *Pinus morrisonicola* Hayata as a promising source for the development of cosmetics. In order to further explore the active components, twenty compounds were isolated from the extract, including three new compounds 15β-12,13-Dihydroxy-14,15-ethoxy-14,15-epoxylabd-8(20)-en-21-oic acid (**18**) 15α-12,13-Dihydroxy-14,15-ethoxy-14,15-epoxylabd-8(20)-en-21-oic acid (**19**), and Morrisonicolene (**20**). The purified potential active flavonoids showed significant inhibition of MMP-2, consistent with the results of PML. However, in the case of Pro MMP-2 and Pro MMP-9, only compound **8** displayed notable inhibition of Pro MMP-2, whereas PMLEF both showed significant inhibitory effects. This may be attributed to potential synergistic effects, which require further investigation in future studies.

## Figures and Tables

**Table 1:** ^13^C-NMR (125 MHz, Chloroform-d), 1H-NMR (500 MHz, Chloroform-d) data for PML18, 19, 20.

	PML18		PML19		PML20	
	^13^C-NMR	^1^H-NMR	^13^C-NMR	^1^H-NMR	^13^C-NMR	^1^H-NMR
Position	δ_C_	δ_H_ (Multiplet, *J* in Hz)	δ_C_	δ_H_ (Multiplet, *J* in Hz)	δ_C_	δ_H_ (Multiplet, *J* in Hz)
1	39.2	1.07 (1H, m), 1.81 (1H, m)	39.0	1.06 (1H, m), 1.89 (1H, m)	39.0	1.04 (1H, m), 1.08 (1H, m)
2	17.4	1.52 (1H, m), 1.78 (1H, m)	17.2	1.35 (1H, m), 1.74 (1H, m)	20.6	1.45 (1H, m), 1.92 (1H, m)
3	38.2	1.01 (1H, m), 2.12 (1H, m)	38.0	1.03 (1H, m), 2.13 (1H, m)	42.4	1.94 (1H, m), 1.97 (1H, m)
4	44.4	-	44.2	-	44.2	-
5	56.6	1.28 (1H, m)	56.3	1.29 (1H, m)	51.8	1.39 (1H, d, *J =* 4.3 Hz)
6	26.1	1.83 (1H, m), 1.96 (1H, m)	26.0	1.86 (1H, m), 1.95 (1H, m)	25.5	2.20 (1H, m), 2.52 (1H, m)
7	38.9	1.84 (1H, m), 2.36 (1H, m)	38.6	1.86 (1H, m), 2.37 (1H, m)	127.8	5.68 (1H, d, *J =* 6.3 Hz)
8	147.9	-	147.7	-	134.6	-
9	56.8	1.51 (1H, m)	56.7	1.52 (1H, m)	56.2	1.87 (1H, m)
10	40.8	-	40.6	-	37.7	-
11	20.1	1.47 (1H, m), 1.83 (1H, m)	19.9	1.50 (1H, m), 1.85 (1H, m)	21.2	1.45 (1H, m), 1.75 (1H, m)
12	33.0	1.44 (1H, m), 1.75 (1H, m)	32.7	1.31 (1H, m), 1.96 (1H, m)	36.4	1.87 (1H, m), 2.07 (1H, m)
13	81.5	-	81.4	-	79.2	-
14	80.5	3.91 (1H, d, *J =* 4 Hz)	80.5	3.94 (1H, d, *J =* 4 Hz)	42.5	2.35 (1H, d, *J* = 10.7 Hz), 2.81 (d, *J* = 10.7 Hz)
15	109.2	4.96 (1H, d, *J =* 4 Hz)	109.0	4.96 (1H, d, *J =* 4 Hz)	175.7	-
16	107.3	4.82 (1H, s)	106.8	4.83 (1H, s)	90.2	4.18 (1H, dd, *J =* 12.2, 2.8 Hz)
17	62.7	3.51 (1H, m), 3.77 (1H, m)	64.5	3.52 (1H, m), 3.78 (1H, m)	41.4	2.30 (1H, m), 2.40 (1H, m)
18	15.4	1.18 (3H, s)	14.9	1.20 (3H, s)	178.9	-
19	63.3	3.45 (1H, m), 3.74 (1H, m)	63.0	3.47 (1H, m), 3.77 (1H, m)	29.5	1.20 (3H, s)
20	15.4	1.18 (3H, s)	14.9	1.20 (3H, s)	14.4	0.75 (3H, s)
21	107.6	4.55 (1H, brs), 4.80 (1H, brs)	106.9	4.64 (1H, brs), 4.81 (1H, brs)	-	-
22	183.3	-	182.6	-	-	-
23	29.2	1.20 (3H, s)	29.0	1.22 (3H, s)	-	-
24	13.0	0.57 (3H, s)	12.7	0.59 (3H, s)	-	-

**Table 2:** Main effects of isolation compound from *Pinus morrisonicola* on cosmeceutical activity.

Number	Compound Name	Cosmeceutical Activity	Reference
PML1	Chrysin	Anti-photoaging, Anti-melanogenesis	[32]
PML2	Apigenin	Anti-UV radiation, Anti-aging,	[33,34]
		Anti-allergic, Anti-inflammatory	
PML3	Kaempferol 3-*O*-(6″-*O*-E- coumaroyl)-β-d-glucopyranoside	Antioxidant, Anti-inflammatory	[41]
PML4	Kaempferol 3-*O*-(6″-*O*-E-feruloyl)-β-d-glucopyranoside	Antioxidant	[38]
PML5	Kaempferol 3-*O*-(3″, 6″-di-*O*-E-p-coumaroyl)-β-d-glucopyranoside	Antioxidant	[39]
PML6	Stenopalustrosides C	-	-
PML7	Kaempferol 3-*O*-(5″-*O*-Z-p-coumaroyl)-α-l-arabinofuranoside	-	-
PML8	Kaempferol 3-*O*-(5″-*O*-E-p-coumaroyl)-α-l-arabinofuranoside	-	-
PML9	Kaempferol 3- *O*-(5″-*O*-E-feruloyl)-α-l-arabinofuranoside	-	-
PML10	Pinoresinol	Antioxidant, Anti-UV radiation	[37]
PML11	Matairesinol	Antioxidant	[40]
PML12	7-Hydroxycoumarin	-	-
PML13	Loliolide	Anti-aging, Photoprotective	[35,36]
PML14	Benzeneacetic acid	-	-
PML15	3-Acetoxylabda-8(20),13-diene-15-oic acid	-	-
PML16	3-Hydroxylabda-8(20),13-diene-15-oic acid	-	-
PML17	13-Labdadien-16, 15-olid-18-oic acid	-	-
PML18	15β,16β-Diethoxy,13,14-dihydroxy-labd-8(21)-en-22-oic acid	-	-
PML19	15β,16α-Diethoxy,13,14-dihydroxy-labd-8(21)-en-22-oic acid	-	-
PML20	Morrisonicolene	-	-

## Data Availability

Not applicable.

## Supplementary Materials

The following supporting information can be downloaded at: https://www.mdpi.com/article/10.3390/molecules28135063/s1, Figure S1. PML18 ^1^H-NMR spectrum. Figure S2. PML18 DEPT-NMR spectrum. Figure S3. PML18 ^1^H-^1^H COSY NMR spectrum. Figure S4. PML18 ^1^H-^1^H NOESY NMR spectrum. Figure S5. PML18 ^1^H-^13^C HSQC NMR spectrum. Figure S6. PML18 ^1^H-^13^C HMBC NMR spectrum. Figure S7. PML19 ^1^H-NMR spectrum. Figure S8. PML19 ^13^C-NMR spectrum. Figure S9. PML19 ^1^H-^1^H NOESY NMR spectrum. Figure S10. PML20 ^1^H-NMR spectrum. Figure S11. PML20 DEPT-NMR spectrum. Figure S12. PML20 ^1^H-^1^H COSY NMR spectrum. Figure S13. PML20 ^1^H-^1^H NOESY NMR spectrum. Figure S14. PML20 ^1^H-^13^C HSQC NMR spectrum. Figure S15. PML20 ^1^H-^13^C HMBC NMR spectrum. Figure S16. PML18 LC-MS spectrum. Figure S17. PML19 LC-MS spectrum. Figure S18. PML20 LC-MS spectrum. Figure S19. Cytotoxicity of compounds **3**, **4**, **5**, **7** and **8** to HT-1080 cells. Results of cytotoxicity of each sample were expressed as % of control cells and mean ± SD (*n* = 3). *p*-values were derived from one-way ANOVA with Dunnett’s multiple comparison tests. * *p*-value < 0.05. Table S1. Table of PML18 and PML20 HMBC spectrum.

## Sample Availability

Samples of the compound **18**, **19**, and **20** is available from the authors.
