# Supplementary material for: Anti-Aging Constituents from Pinus morrisonicola Leaves
Source: Molecules. 2023 Jun 28;28(13):5063. doi: 10.3390/molecules28135063 (PMC10343237; doi:10.3390/molecules28135063)
Supplement: Supplementary file 1 [file molecules-28-05063-s001.zip › molecules-2424598-supplementary.pdf]

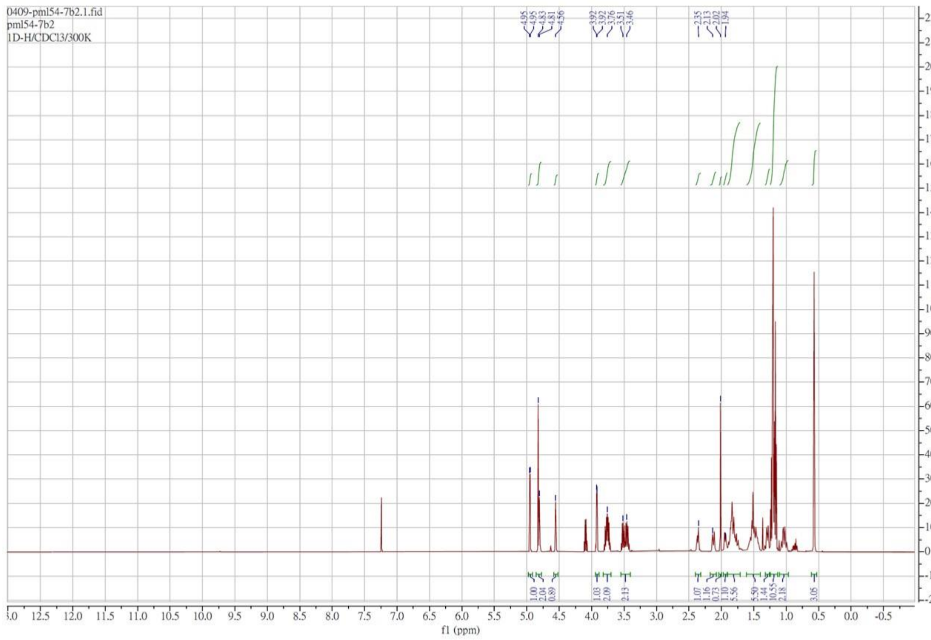

Figure S1. PML18 ^{1}H-NMR spectrum.

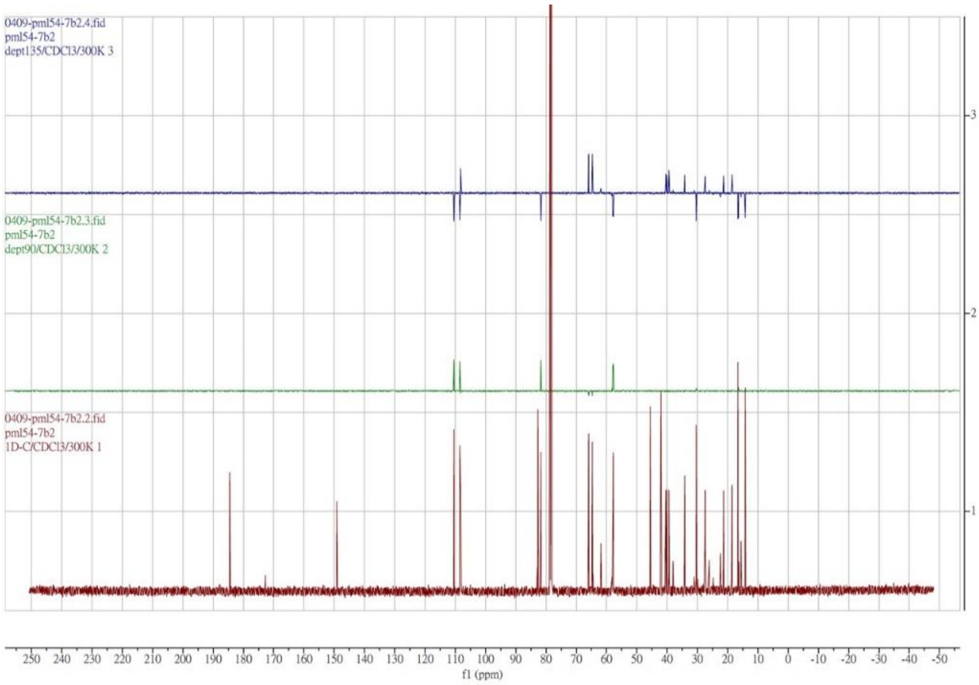

Figure S2. PML18 DEPT-NMR spectrum.

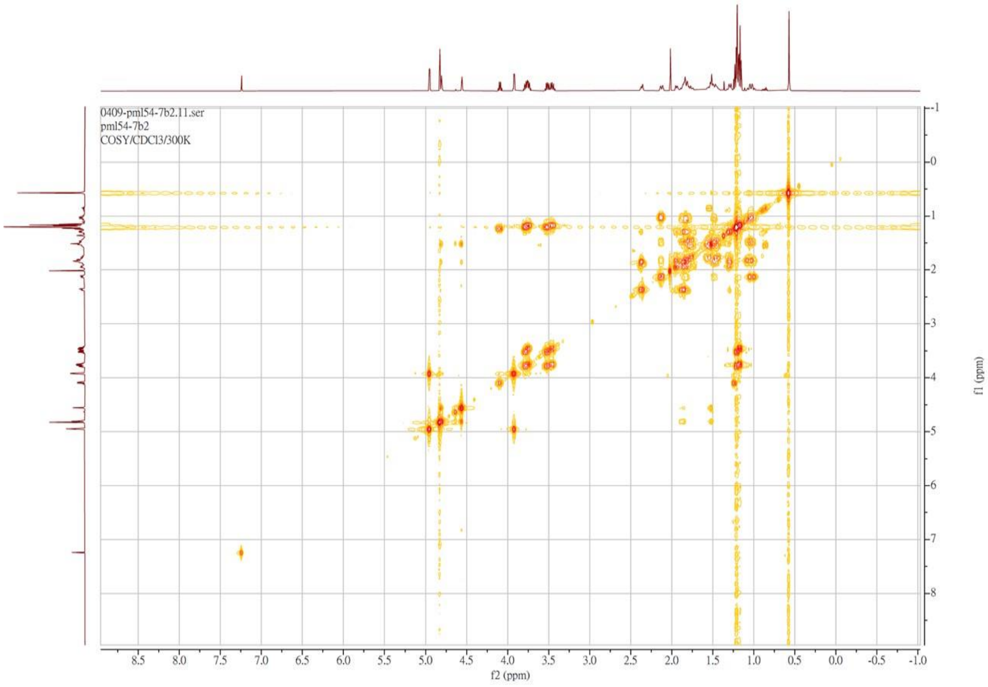

Figure S3. PML18 ^1H-^1H COSY NMR spectrum.

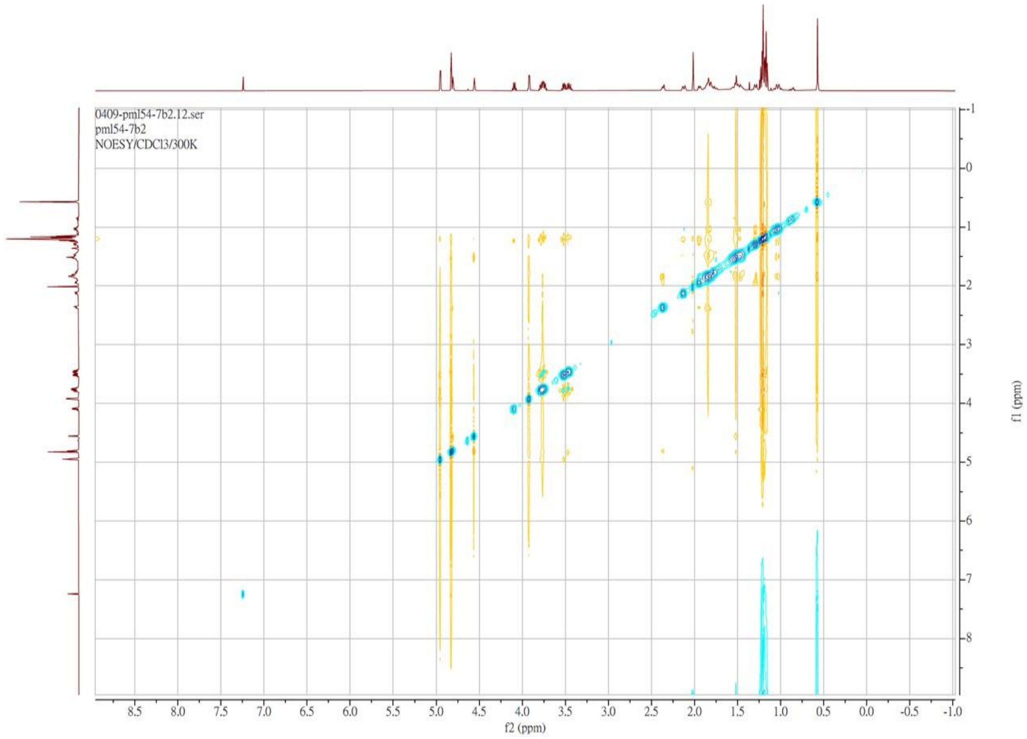

Figure S4. PML18 ^1H-^1H NOESY NMR spectrum.

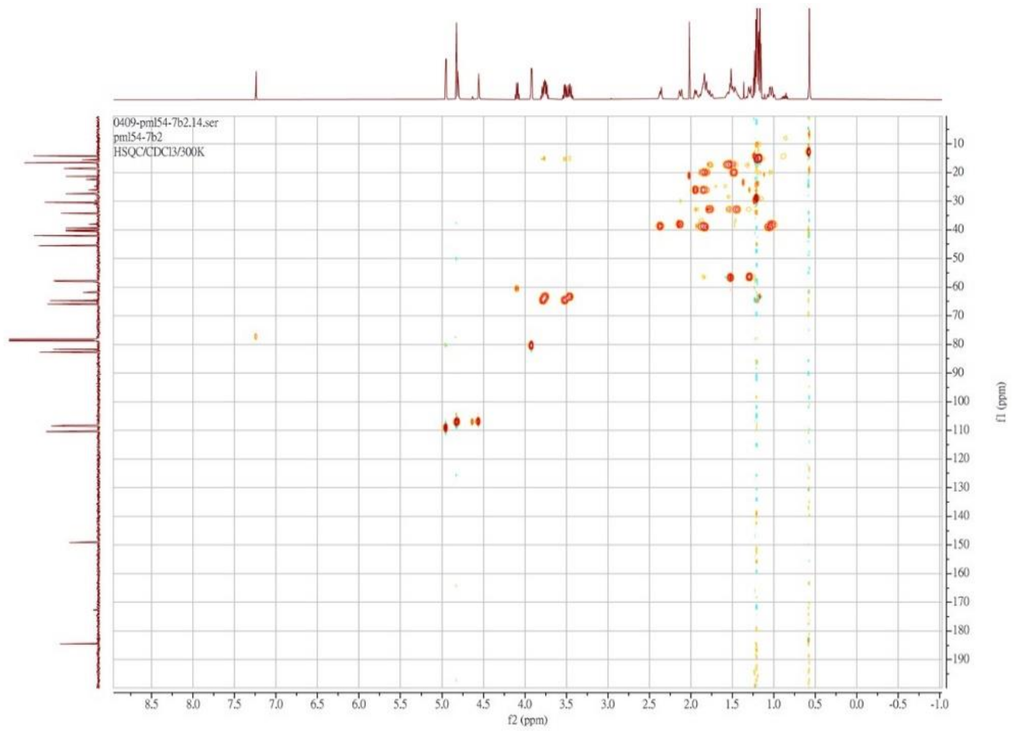

Figure S5. PML18 ^{1}H-^{13}C HSQC NMR spectrum.

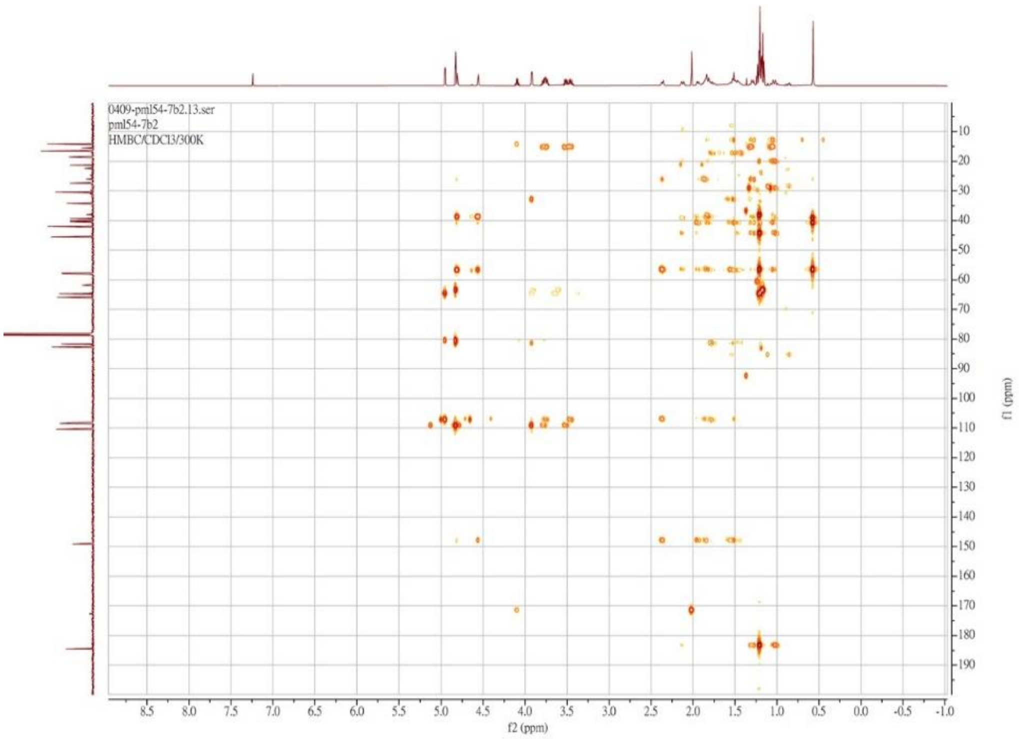

Figure S6. PML18 ^{1}H-^{13}C HMBC NMR spectrum.

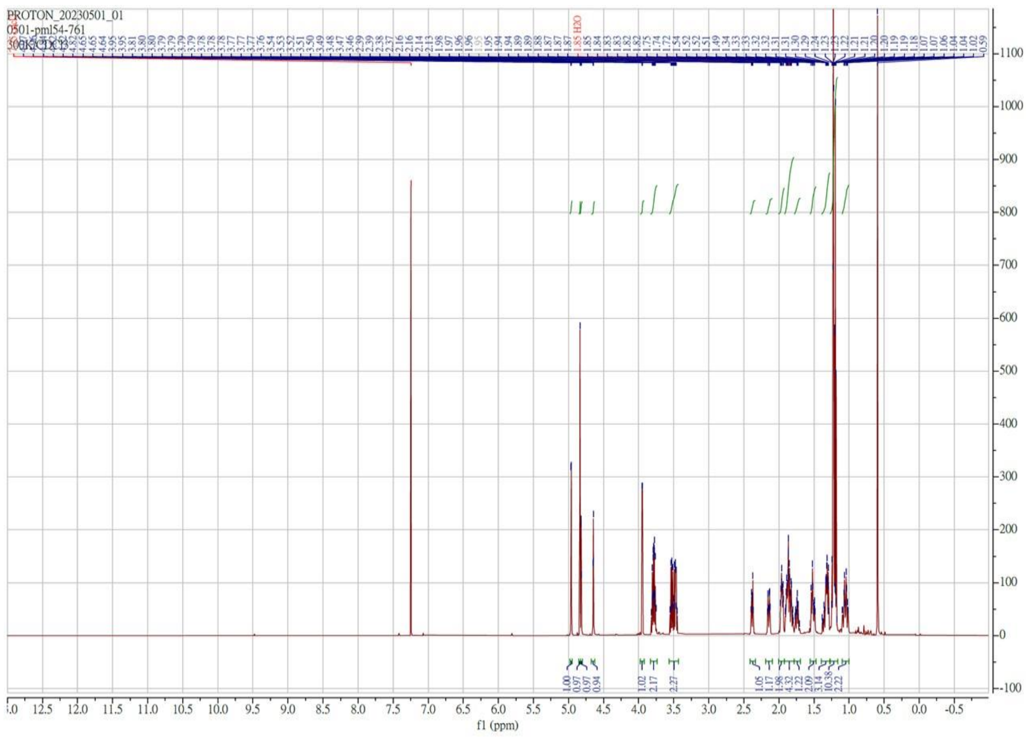

Figure S7. PML19 ^{1}H-NMR spectrum.

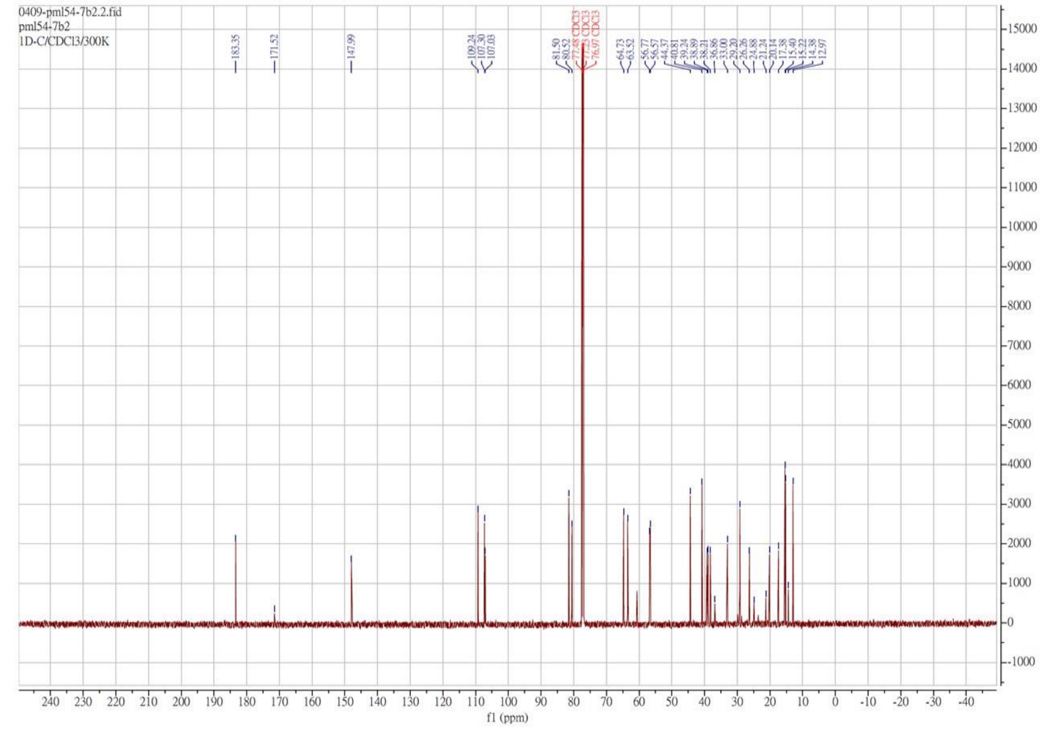

Figure S8. PML19 ^{13}C-NMR spectrum.

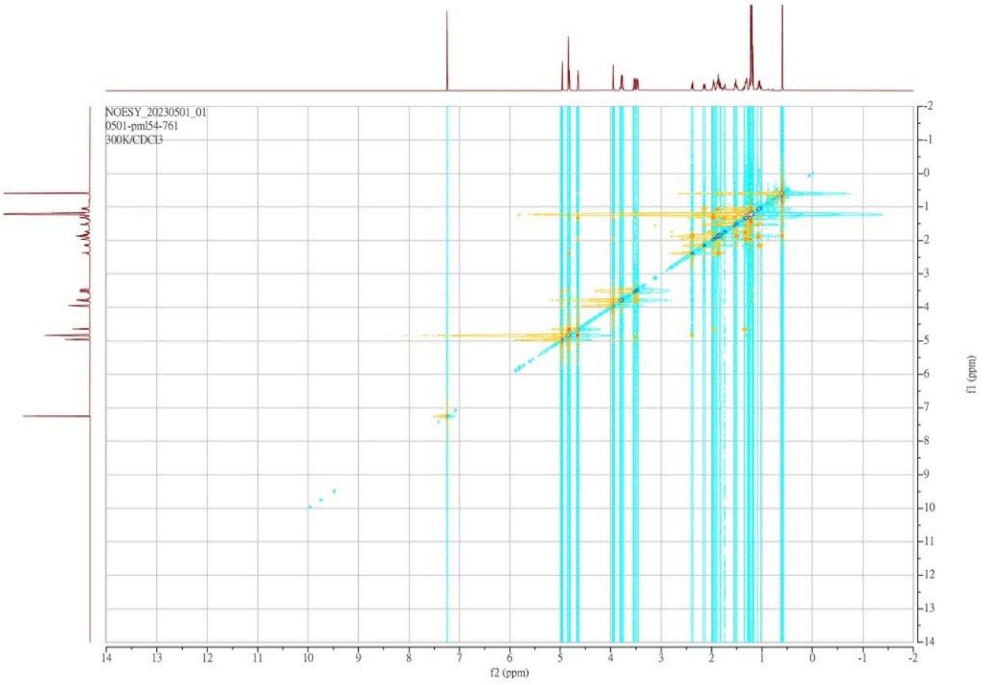

Figure S9. PML19 ^{1}H-^{1}H NOESY NMR spectrum.

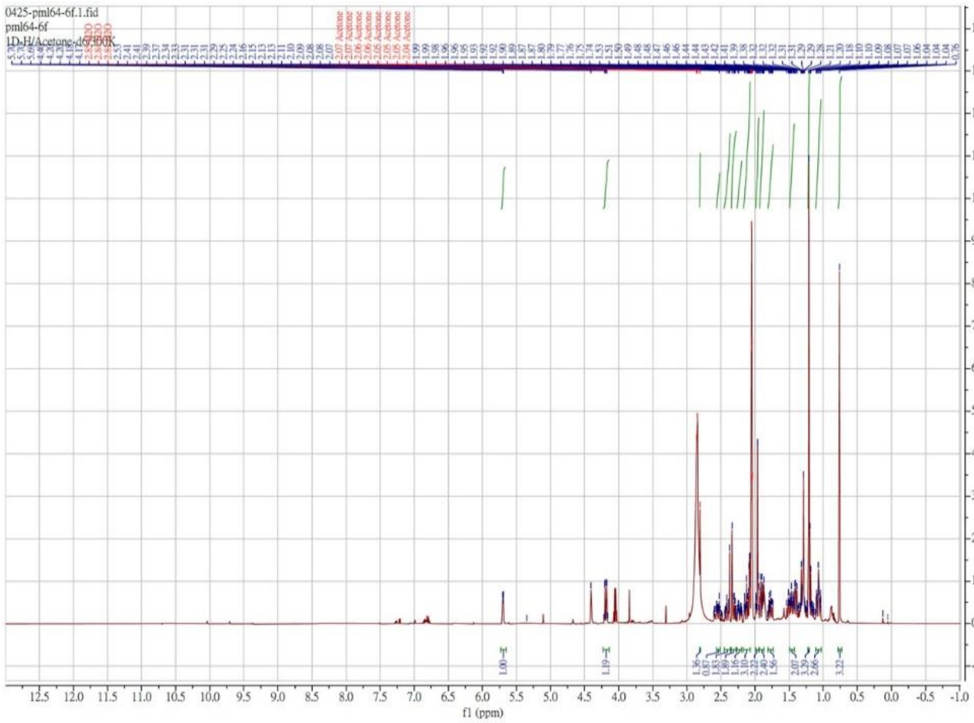

Figure S10. PML20 ^{1}H-NMR spectrum.

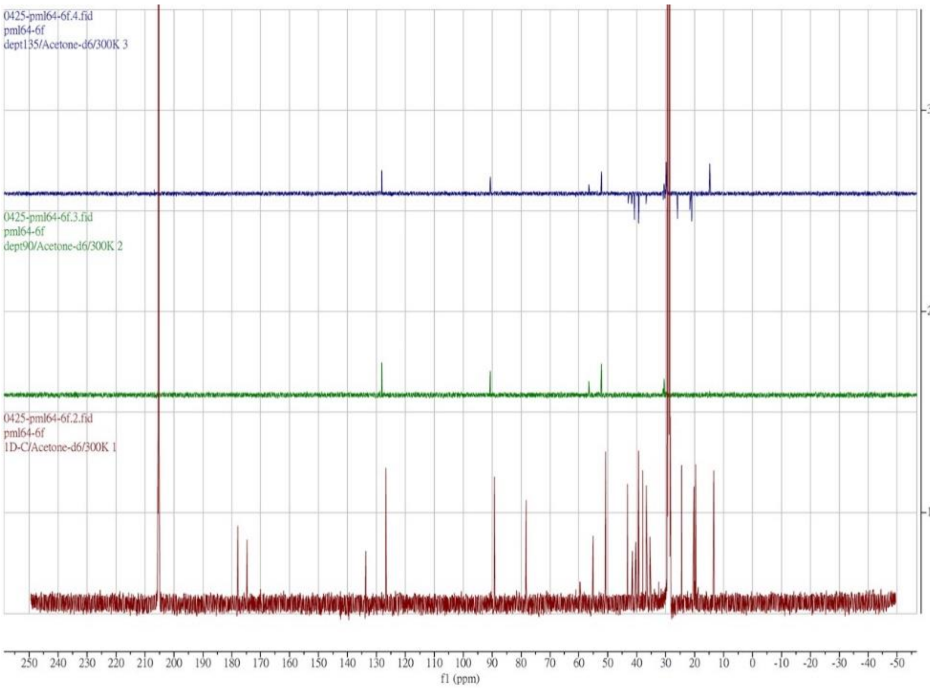

Figure S11. PML20 DEPT-NMR spectrum.

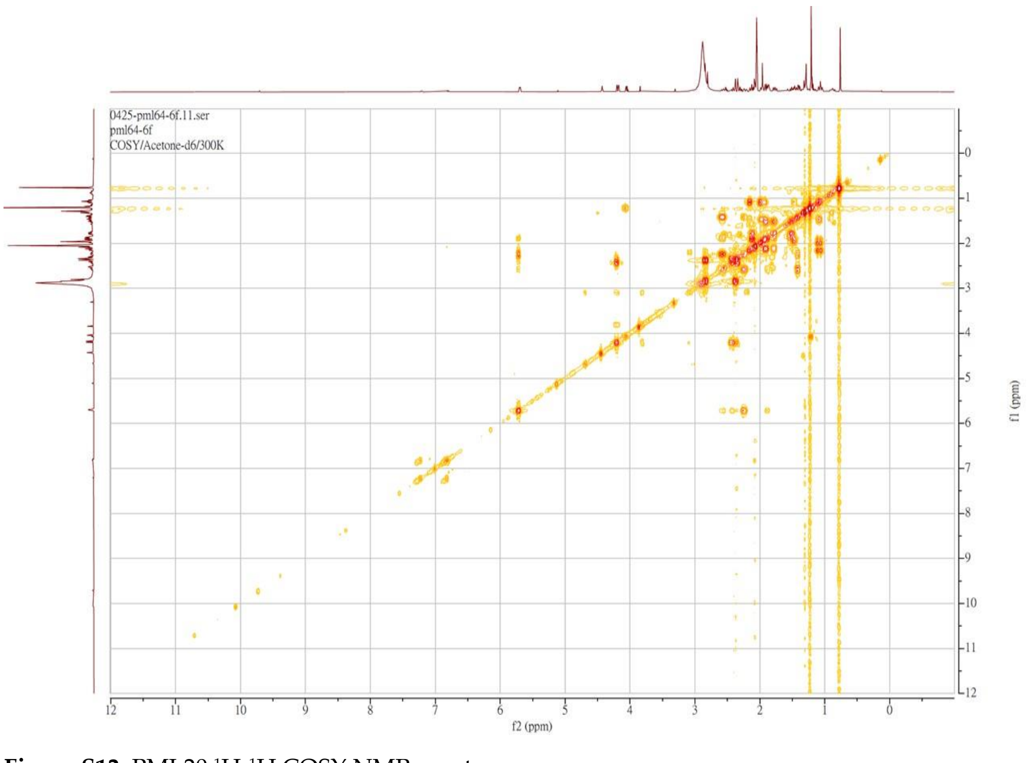

Figure S12. PML20 ^{1}H-^{1}H COSY NMR spectrum.

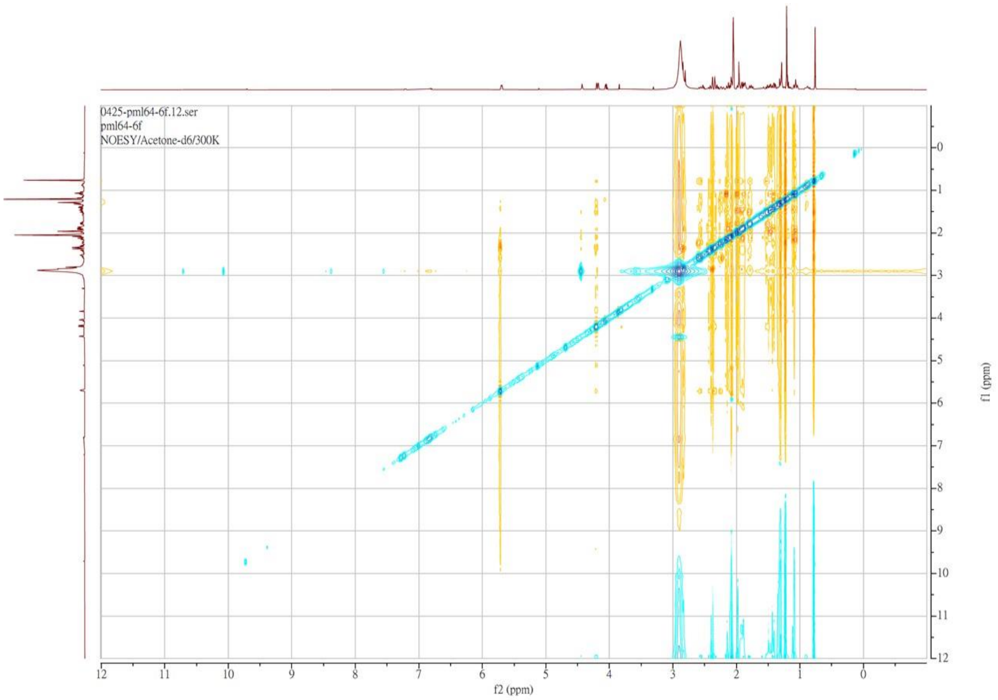

Figure S13. PML20 ^{1}H-^{1}H NOESY NMR spectrum.

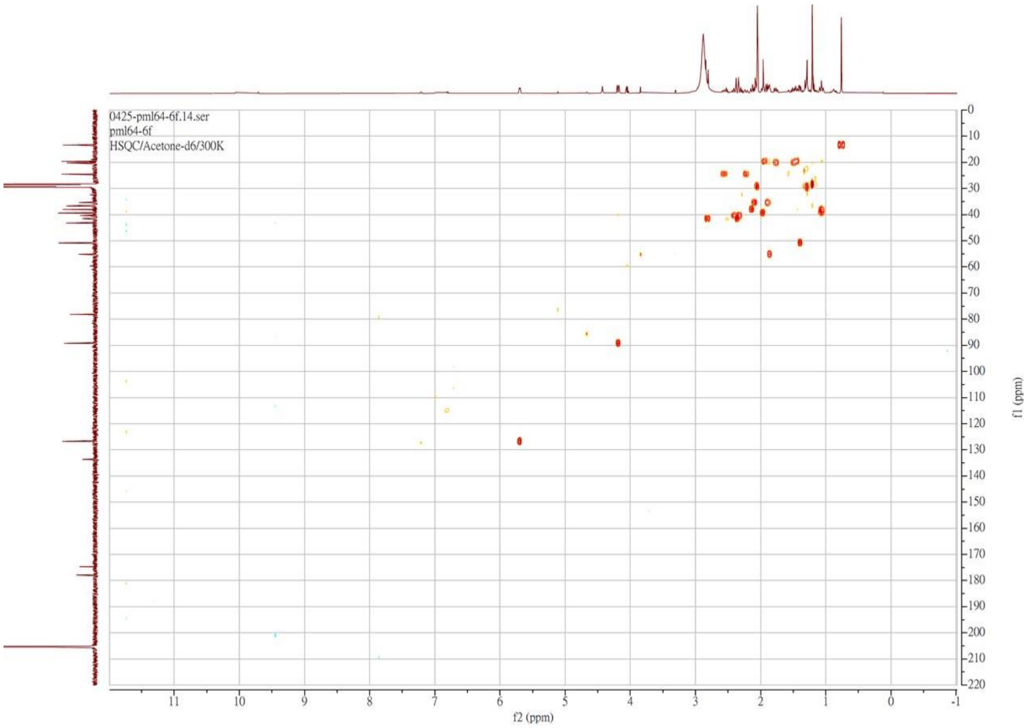

Figure S14. PML20 ^{1}H-^{13}C HSQC NMR spectrum.

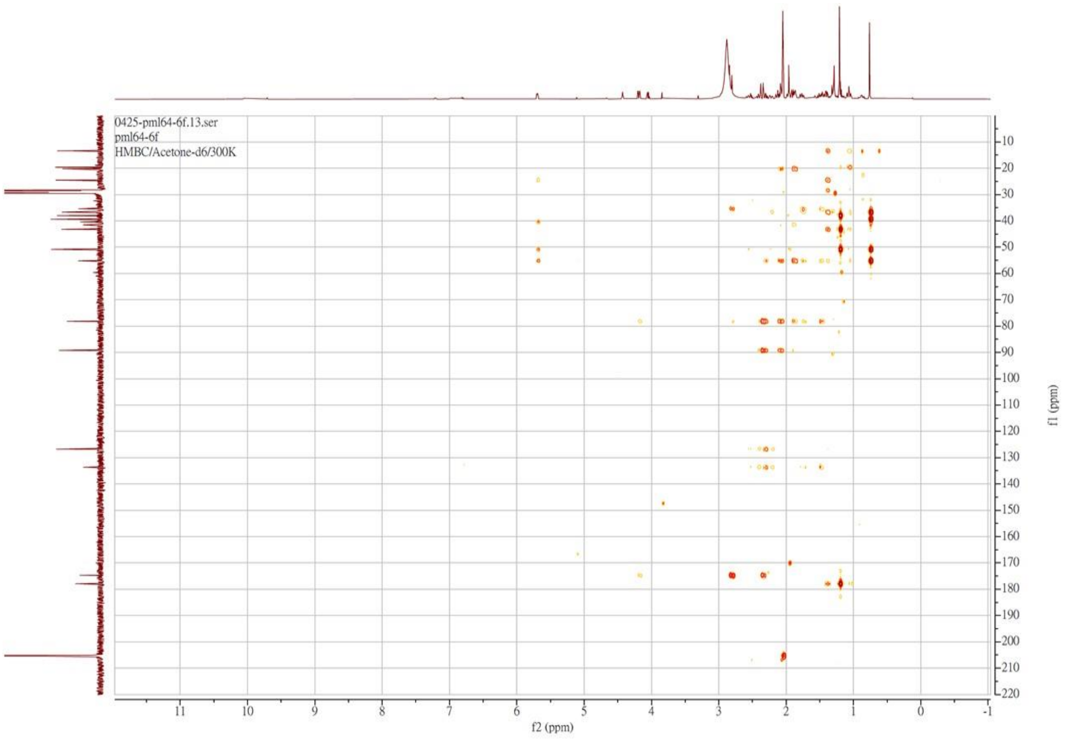

Figure S15. PML20 ^{1}H-^{13}C HMBC NMR spectrum.

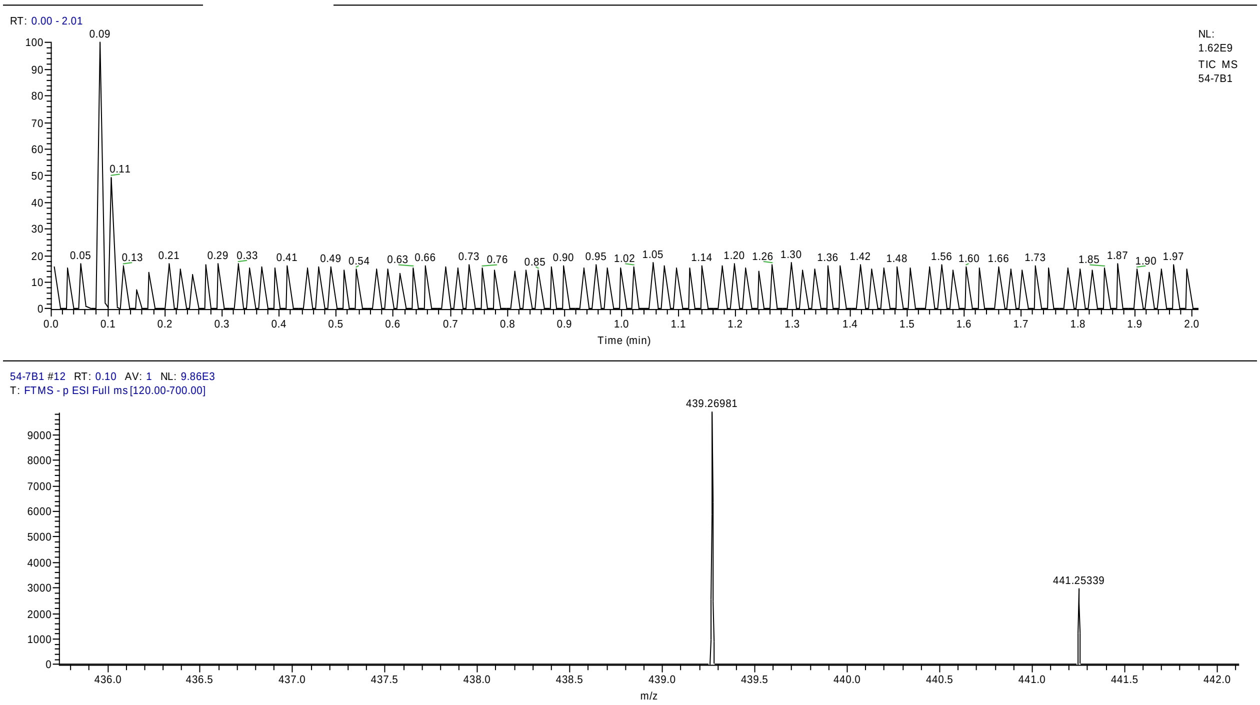

Figure S16. PML18 LC-MS spectrum.

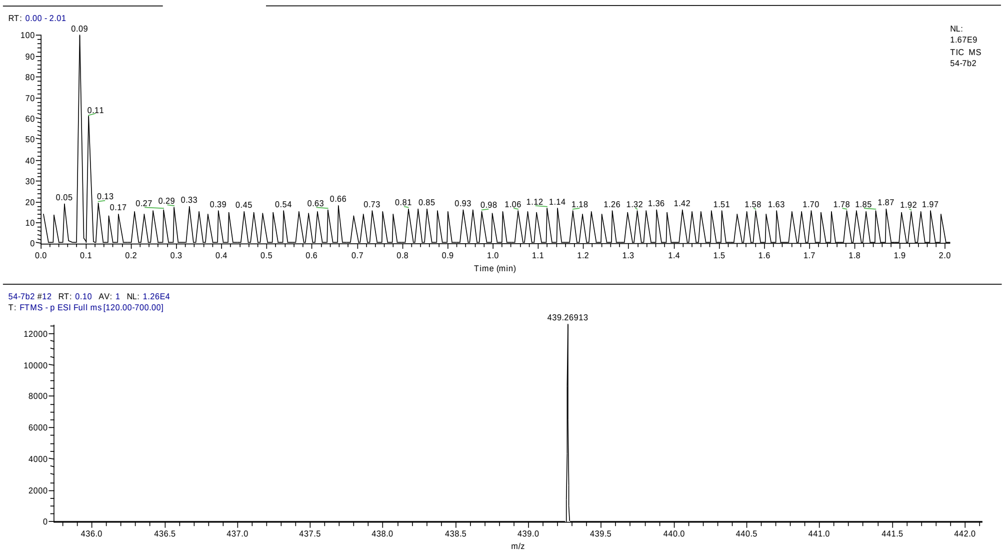

Figure S17. PML19 LC-MS spectrum.

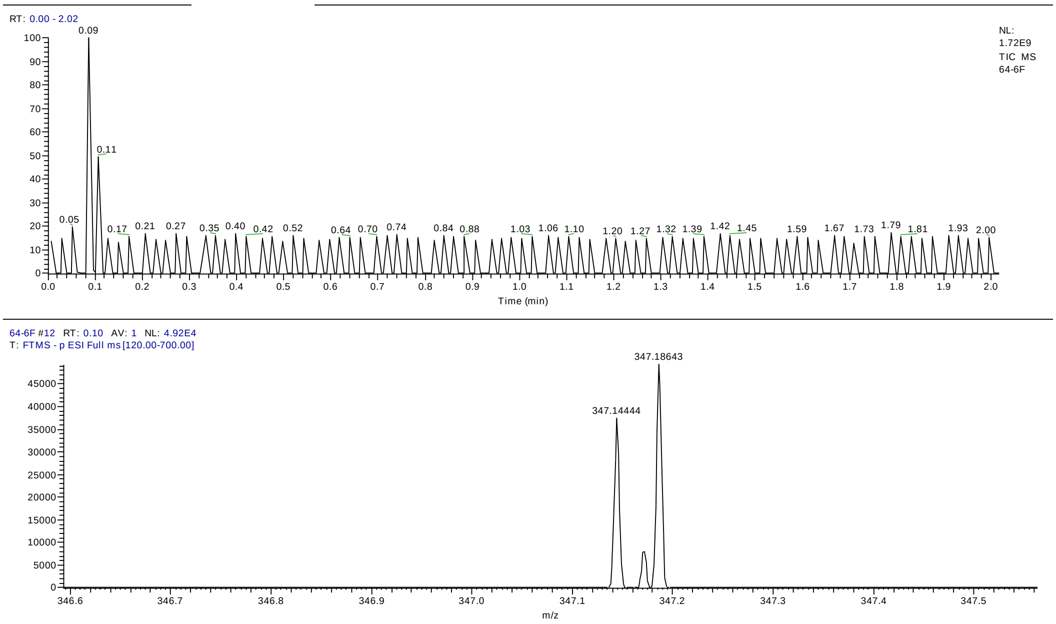

Figure S18. PML20 LC-MS spectrum.

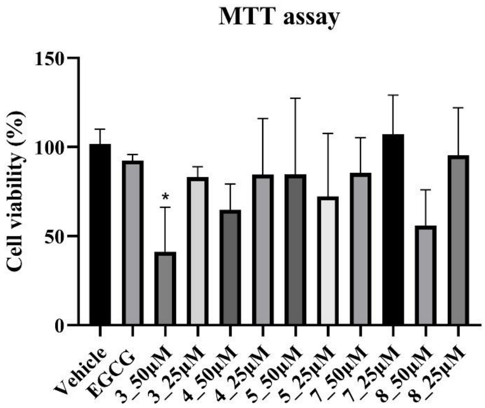

Figure S19. Cytotoxicity of compounds **3**, **4**, **5**, **7** and **8** to HT-1080 cells. Results of cytotoxicity of each sample were expressed as % of control cells and mean ± SD (n = 3). p-values were derived from one-way ANOVA with Dunnett's multiple comparison tests. * *p*-value < 0.05.

Table S1. Table of PML18 and PML20 HMBC spectrum.

	PML18		PML20	
	^{1}H-NMR	HMBC	^{1}H-NMR	HMBC
**Position**	δ_H (Multiplet , *J* in Hz)	δ_C	δ_H (Multiplet , *J* in Hz)	δ_C
1	1.07(m), 1.81(m)	40.8, 56.6	1.04(m), 1.08(m)	14.4, 20.6, 37.7, 42.4, 51.8
2	1.52(m), 1.78(m)	13.0, 40.8, 56.6,	1.45(m), 1.92(m)	37.7, 51.8, 178.9
3	1.01(m), 2.12(m)	20.1, 29.2, 44.4, 56.6,183.3	1.94(m), 1.97(m)	20.6, 51.8
4	---	---	---	---
5	1.28(m)	13.0, 26.1, 40.8, 44.4, 183.3	1.39(d, *J* = 4.3 Hz)	14.4, 25.5, 29.5, 37.7, 44.2, 56.2, 178.9
6	1.83(m), 1.96(m)	38.9,40.8, 56.6, 147.9	2.20(m), 2.52(m)	37.7, 127.8, 134.6
7	1.84(m), 2.36(m)	26.1, 56.6, 107.6, 147.9	5.68(d, *J* = 6.3 Hz)	25.5, 41.4, 51.8, 56.2
8	---	---	---	---
9	1.51(m)	13.0, 33.0,40.8, 56.6, 81.5, 147.9	1.87(m)	21.2, 41.4, 79.2, 90.2
10	---	---	---	---
11	1.47(m), 1.83(m)	38.9, 40.8, 56.8,107.6, 147.9	1.45(m), 1.75(m)	14.4, 36.4, 56.2, 79.2,
12	1.44(m), 1.75(m)	56.8, 81.5, 107.3	1.87(m), 2.07(m)	21.2, 41.4, 56.2, 79.2, 90.2,
13	---	---	---	---
14	3.91(d, *J* = 4 Hz)	33.0, 81.5, 109.2	2.35(d, *J* = 10.7 Hz), 2.81(d, *J* = 10.7 Hz)	36.4, 79.2, 90.2, 175.7
15	4.96(d, *J* = 4 Hz)	62.7, 80.5, 107.3	---	---
16	4.82(s)	63.3, 81.5, 109.2	4.18(dd, *J* = 12.2 , 2.8 Hz)	79.2, 175.7
17	3.51(m), 3.77(m)	15.4, 109.2	2.30(m), 2.40(m)	56.2, 79.2, 90.2, 127.8, 134.6, 175.7
18	1.18(s)	62.7	---	---
19	3.45(m), 3.74(m)	15.4, 107.3	1.20(s)	42.4, 51.8, 178.9
20	1.18(s)	63.3	0.75(s)	37.7, 39.0, 51.8, 56.2
21	4.55(brs), 4.80(brs)	38.9, 56.8, 147.9	---	---
22	---	---	---	---
23	1.20(s)	38.2, 44.4, 56.6, 183.3	---	---
24	0.57(s)	39.2, 40.8, 56.6, 56.8	---	---
